# Calcifying Epithelial Odontogenic Tumour. Review of The Literature and Own Experience

**DOI:** 10.34763/devperiodmed.20192301.3438

**Published:** 2019-04-08

**Authors:** Tomasz Piskadło, Robert Brodowski, Mariusz Książek, Paweł Pakla, Mateusz Dymek, Piotr Haberko, Jan Franczak, Wojciech Stopyra, Bogumił Lewandowski

**Affiliations:** 1 Department of Maxillofacial Surgery, F. Chopin Clinical Voivodeship Hospital in Rzeszow, Poland; 2 Department of Pathology, F. Chopin Clinical Voivodeship Hospital in Rzeszow, Poland; 3 Chair of Emergency Medical Services, Faculty of Medicine, University of Rzeszow, Poland

**Keywords:** Calcifying epithelial odontogenic tumour, CEOT, Pindborg tumour, calcifying ameloblastoma, wapniejący nabłonkowy guz zębopochodny, CEOT, guz Pindborga, calcyfying ameloblastoma

## Abstract

The present paper discusses the case of a patient who was surgically treated for a rare calcifying epithelia/ odontogenic tumour (Pindborg tumour) at the Department of Maxillofacia/ Surgery, F. Chopin Clinical Voivodeship Hospital in Rzeszow. Ca/cifying Epithelia/ Odontogenic Tumour (CEOT) is a benign odontogenic tumour arising from the remnants of the dental lamina epithelium. The first three cases of this tumour were recognized by the Danish patho/ogist J.J. Pindborg in 1955. Since then Ca/cifying Epithelia/ Odontogenic Tumour has been commonly referred to as the Pindborg tumour. This type of neop/asm is relatively rare, since it occurs in approximately 0.4% of all odontogenic tumour cases. Due to a fairly common tendency of recurrence, estimated to be approximately 14% of all cases, the preferred o choice of treatment is radical surgical procedure and postoperative follow-up. Appropriate clinical and histopatho/ogical diagnosis is very important before applying the most suitable surgical treatment. Based on the case reviewed and the available literature, we can confirm the suitability of the therapeutic procedure course aligned with contemporary views, guidelines and established standards.

## Streszczenie

Przedstawiono przypadek pacjentki leczonej chirurgicznie z powodu nabłonkowego wapniejącego guza zębopochodnego w Klinice Chirurgii Szczękowo-Twarzowej Klinicznego Szpitala Wojewódzkiego nr 1 w Rzeszowie. Nabłonkowy wapniejący guz zębopochodny to miejscowo złośliwy nowotwór zębopochodny wywodzący się z pozostałości nabłonka listewki zębowej. Po raz pierwszy 3 przypadki tego guza w 1955 roku opisał duński patolog J.J. Pindborg. Od tego momentu CEOT określany jest potocznie guzem Pindoborga. Guz ten występuje stosunkowo rzadko, bo w około 0,4% wszystkich przypadków guzów zębopochodnych. Ze względu na dość częstą tendencję do nawrotów, określaną na około 14% przypadków jego leczeniem z wyboru jest radykalny zabieg chirurgiczny i systematyczna kontrola pooperacyjna. W pracy przedstawiono przypadek pacjentki leczonej chirurgicznie w Klinice Chirurgii Szczękowo-Twarzowej Klinicznego Szpitala Wojewódzkiego im. F. Chopina w Rzeszowie z powodu rzadko występującego nabłonkowego wapniejącego guza zębopochodnego CEOT (guz Pindborga). Bazując na naszym przypadku oraz dostępnej wiedzy możemy potwierdzić słuszność obecnych wytycznych oraz standardów postępowania

## Introduction

Calcifying Epithelial Odontogenic Tumour (CEOT) was first recognized as a separate disease entity in 1955 by the Danish pathomorphologist Jens Jorgen Pindborg [1,2,4]. According to contemporary views, this tumour is considered to be a developmental disorder, arising from the remnants of the dental lamina epithelium [1, 2, 3]. Currently, the name Pindborg tumour is a synonym for CEOT in the scientific literature, even though only CEOT is acceptable in the World Health Organisation (WHO) odontogenic tumour classification [3]. It maybe found in any age group, however, the highest incidence is reported in the third and fourth decade of life and accounts for approximately 0.4% of all odontogenic tumours. Considering the location, the intraosseous (or central) form is more common and manifests in approximately 95% of cases, while the other, extraosseous (or peripheral) form is found in the remaining 5%. The central type affects the mandible, whereas the less common peripheral type develops mainly the anterior segment of the maxilla [2, 5]. The authors emphasise the differences in the histogenesis of the central and peripheral forms of CEOT. The central form is a developmental disorder of the enamel organ epithelium and it is usually associated with an impacted tooth. The peripheral form arises as part of the dental lamina epithelium or it may develop from the basal membrane of the stratified squamous epithelium, which forms the lining of the oral cavity [2,3]. This type of tumour is initially difficult to diagnose, since it does not cause any specific symptoms. Most commonly, it is detected incidentally during a radiological examination due to other reasons. The radiological imaging of the tumour in ordinary x-ray is non-specific, and that is why imaging diagnostics should be supported by a Computed Tomography (CT) scan and/ or Magnetic Resonance Imaging (MRI). The first clinically evident sign of CEOT is the local expansion of the affected bone, migration of the teeth in this area and numbness. The mucosa overlying the tumour becomes inflamed, reddish and swollen, with a tendency to bleed even with the slightest trauma. Due to its clinical manifestation and the expanding growth of the tumour causing local damage to the bone, CEOT is considered to be a locally aggressive, although benign odontogenic tumor, and this determines the method of treatment. According to up-to-date guidelines, the preferred choice of treatment is wide surgical excision. Approximately 14% of these neoplasms may recur locally, which requires a close post-surgical follow-up [1, 2, 3, 5, 6, 7]. In everyday clinical practice it is sometimes difficult to diagnose and differentiate CEOT, and this could cause a potential diagnostic problem for clinicians. As CEOT cases are rarely described in the scientific literature, it appears reasonable to present two of our own cases.

## Objective

The aim of this article is to present the incidence, clinical and histopathological diagnosis and treatment of CEOT based on an overview of the literature and our own case of CEOT treated at the Department of Maxillofacial Surgery, E Chopin Clinical Voivodeship Hospital in Rzeszow.

## Case report

**Fig. 1:**
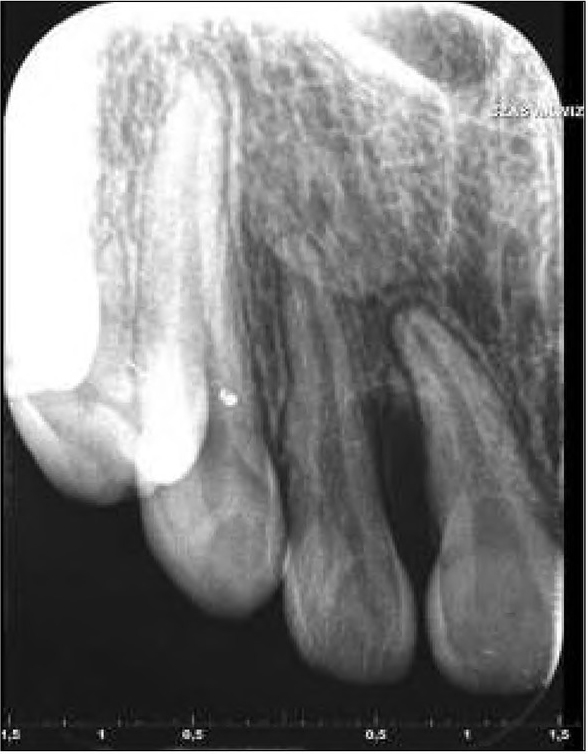
Intraoral x-ray. Bone loss between teeth 11-12. Rye. 1. Zdjęcie zębowe wewnątrzustne. Widoczna utrata kości pomiędzy zębami 11-12.

A 15-year-old patient was referred to the Maxillofacial Surgery Outpatient Clinic due to an epulis-like tumour localized on the alveolar process of the maxilla adjacent to teeth 11 and 12. The patient claimed that the lesion had appeared a few months before. She denied any other symptoms apart from discomfort and swelling in that area. Clinical examination revealed an exophytic gingival tumour, with a wide base, between teeth 11 and 12, firm texture, painless, overlying normal-appearing mucosa. During the first visit, an intraoral x-ray of the affected area was performed, which revealed damage to the bone of the alveolar process between teeth 11-12 (fig. 1).

**Fig. 2:**
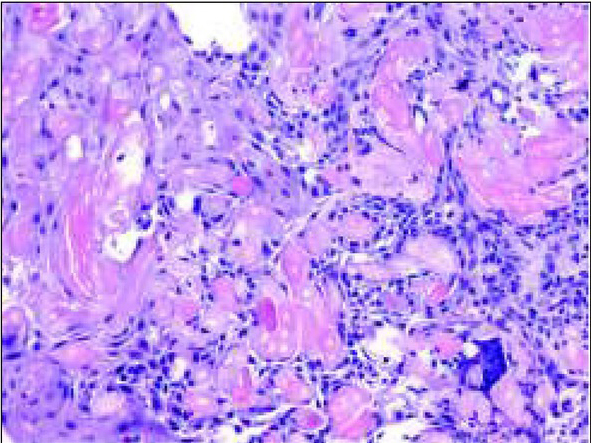
H&E stain, objective x 20. Sheets of large polyhedral epithelial cells with bright, eosinophilic cytoplasm. Cells exhibit marked pleomorphism, however no mitotic figures are seen. There are few areas with acellular bright eosinophilic amyloid-like material. Ryc. 2. Barwienie hematoksylina i eozyna, obiektyw x20. Widoczne płaty wielobocznych komórek nabłonkowych z jasną, eozynochłonną cytoplazmą. Pomimo że komórki wykazują duży pleomorfizm komórkowy, nie uwidoczniono figur podziałów mitotycznych. Widoczne obszary bezpostaciowej, amyloidopodobnej eozynochłonnej substancji.

**Fig. 3:**
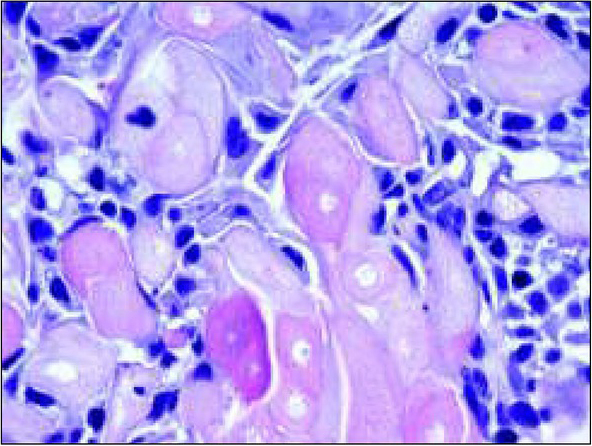
H&E, objective x 63. This high magnification picture shows in detail the large eosinophilic cells as well as the collection of amyloid-like material. Ryc. 3. Barwienie hematoksylina i eozyna, obiektyw x63. Na tym dużym powiększeniu widoczne są w szczegółach komórki z eozynochłonną cytoplazmą oraz amyloidopodobna substancja.

**Fig. 4:**
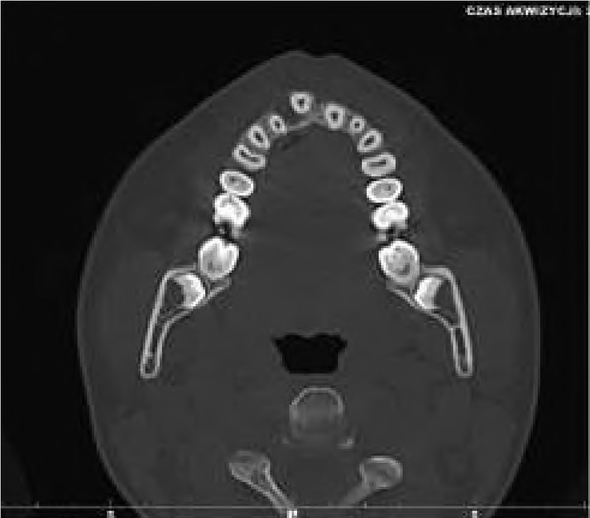
CT scan, osteolytic defect in the alveolar process. Ryc. 4. Skan TK. Zniszczenie struktury kostnej wyrostka zębodołowego.

**Fig. 5:**
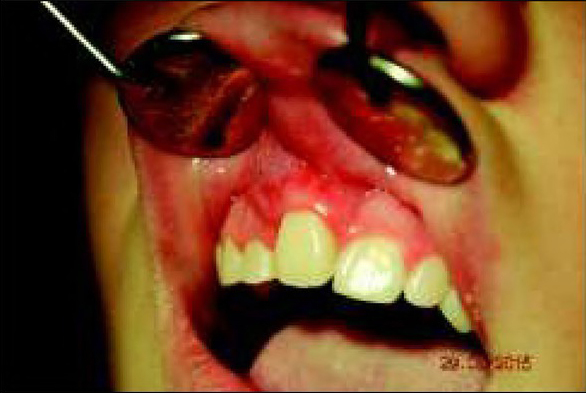
Extraoral photo. Migration towards the front of tooth number 11. Ryc. 5. Zdjęcie zewnątrzustne. Przemieszczenie doprzednie zęba 11.

**Fig. 6:**
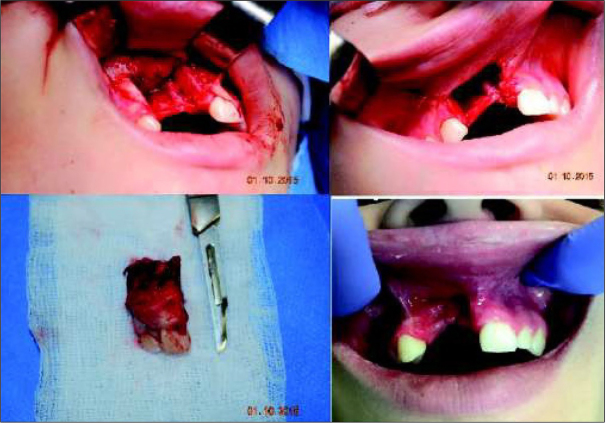
Operation photos. Partial resection of the alveolar process. Ryc. 6. Zdjęcia śródoperacyjne. Częściowa resekcja wyrostka zębodołowego.

**Fig. 7:**
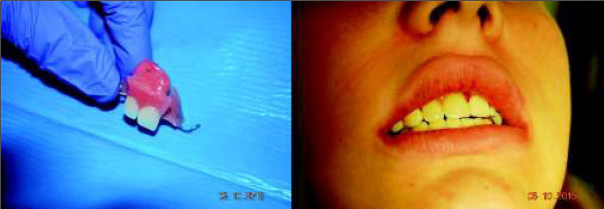
Post-op obturator prosthesis. Ryc. 7. Obturator.

Due to the benign and slow-growing development of the tumour, the patient was enrolled for an excisional biopsy under local anaesthesia. Surgical procedure was performed and the surgical specimen was sent for a histopathological examination. Surgery was uneventful and the wound healed without complications. Histopathological examination of this primary excisional biopsy was performed at the hospital Pathology Department, and the specimen received for examination consisted of 4 small tissue fragments measuring from 3 to 13mm. The microscopic examination revealed islands and sheets of eosinophilic polyhedral epithelial cells (fig. 2 and 3) surrounded by fibrous connective tissue. The epithelial cells exhibited significant cellular and nuclear pleomorphism, however no mitotic activity was seen (fig. 3). Intermixed with the epithelial cells, there were areas with the presence of homogenous eosinophilic, amyloid-like material (fig. 3), and the final diagnosis was calcifying epithelial odontogenic tumour (CEOT). Unfortunately, as the specimen received for examination was fragmented, no comments on the completeness of the procedure could be made based on the microscopic examination. The patient was scheduled for frequent follow-up appointments and a few months later, during a follow-up visit, there was evidence of tumour recurrence. A CT scan was performed and revealed an osteolytic defect measuring 10x8 mm, filled with thick fluid content in the alveolar process of the right maxilla between teeth 11 and 12 (fig. 4). The lesion affected the periapical area of teeth 11-12, causing labial migration of tooth 11 (fig.5). The patient was readmitted to the clinic for a wider local excision surgery. Due to the early onset of the recurrence, the clinical course, and the patient’s young age, she was qualified for a partial resection of the alveolar process affected by the tumour and teeth number 11-12, with the goal to achieve unequivocal clear margins (fig. 6). Following the surgical procedure, before discharge, temporary prosthetic restoration was completed (fig. 7). A histopathological examination confirmed the previous diagnosis of CEOT. Also, as the surgical specimen received for examination consisted of a single fragment composed of bony and fibrotic tissue and measuring approximately 22 x 18 x 26mm, microscopic examination confirmed the completeness of the surgical excision of the lesion. The patient has been closely followed up in the Oral and Maxillofacial Surgery Hospital Outpatient Clinic and there were no signs of local recurrence in a 2-year period. The patient requires periodic replacement of the postoperative prosthesis.

## Discussion

The literature refers to CEOT as a rare, slowly growing tumour, arising from the odontogenic epithelium, representing approximately 0.4% of all odontogenic tumours. It may occur over a wide age range [5], with a good example of the 15-year-old girl described above. Thetumour has no gender predilection and both men and women are susceptible to CEOT. In women it occurs on average 10 years later than in men [2,5]. CEOT occurs in two clinical forms: the more common intraosseous form, accounting for 95% of all cases, and the less common extraosseous (or peripheral) form [2, 5]. Thus far, most of the over 200 described cases of this type of tumour have been associated with the mandible and the intraosseous form. The significantly less common extraosseous (or peripheral) form is most typically seen in the anterior segment of the maxilla [5]. This type of tumour leads to the migration of the adjacent teeth, which is often the first clinical sign. Despite this migration, there is no evidence of teeth loosening or resorption, which is consistent with the observations of other authors. The overlying mucosa often becomes hyperaemic or remains unaltered. Recurrence appears in approximately 14% of the tumour cases [1, 2, 3, 5, 6]. The peripheral form has a lower tendency of recurrence. However, in our case, a recurrence occurred in a short period after surgery. Although the lesions in the mandible involve the central part of the bone, they seldomly cause hypoesthesia in the inferior alveolar nerve. Due to the non-specific radiological imaging of the tumour in a conventional x-ray, it is necessary to extend the diagnostics to a CT scan or an MRI, which provide further information. In a CT image, CEOT appears as a well-defined osteolytic defect of the bone structure. The affected cortical plate of the bone is usually thinner, but rarely leads to tearing. MRI may provide additional diagnostic information, and the tumour in T 2-weighted images is manifested by a hyperintense focus, and in T1-weighted images as a hypointense focus [7]. Most authors claim that the treatment should be individualized, taking into consideration the patient’s age and the location, as well as the extent of the tumour. Small-sized tumours may be removed with a narrow margin, with the recommendation of a strict follow-up, which allows for an early diagnosis of any recurrence - this scenario occurred in the case presented here. Largesized tumours, causing damage to the cortical layer of the bone, with invasion of the surrounding soft tissue, are recommended for block bone resection. Considering the local aggressive nature of the tumour associated with bone involvement, radical surgical treatment, including the resection of the bone, is recommended. In the maxilla it required partial or complete resection, while in the mandible marginal or segmental resection. A partially sparing treatment, with macroscopically unaffected tissue margins, is not justified, as it leads to a short time interval between the operation and the recurrence, as presented in this case. A good treatment result was achieved by a partial resection of the maxillary alveolar process. The patient has had close follow-up care in the Maxillofacial Surgery Clinic for the past 2 years and there has been no evidence of recurrence. Although the post-surgical defect has been partially filled by normal bone tissue, the patient still has to use prosthetic restoration.

## Conclusion

The above-mentioned case confirms the suitability of the therapeutic procedure undertaken, which is aligned with contemporary views, guidelines and established standards. Properly planned and conducted treatment enables therapeutic success and the subsequent prosthetic replacement offers the best aesthetic and functional results.
